# A SERPINE1-Based Immune Gene Signature Predicts Prognosis and Immunotherapy Response in Gastric Cancer

**DOI:** 10.3390/ph15111401

**Published:** 2022-11-14

**Authors:** Xiang Xu, Lipeng Zhang, Yan Qian, Qian Fang, Yongbiao Xiao, Guizeng Chen, Guojing Cai, Alimujiang Abula, Zhao Wang, Ertao Zhai, Jianhui Chen, Shirong Cai, Hui Wu

**Affiliations:** 1 Department of Gastrointestinal Surgery, The First Affiliated Hospital of Sun Yat-sen University, Guangzhou 510080, China; 2 Department of Gastrointestinal Surgery, The Affiliated Kashi Hospital, Sun Yat-sen University, Kashi 844099, China; 3 Department of Gastrointestinal Surgery, The First People’s Hospital of Kashi Prefecture, Kashi 844099, China

**Keywords:** gastric cancer, tumor microenvironment, immune checkpoint inhibitor, immune risk signature, tumor mutation load

## Abstract

Immune checkpoint inhibitors (ICIs) therapy has been successfully utilized in the treatment of multiple tumors, but only a fraction of patients with gastric cancer (GC) could greatly benefit from it. A recent study has shown that the tumor microenvironment (TME) can greatly affect the effect of immunotherapy in GC. In this study, we established a novel immune risk signature (IRS) for prognosis and predicting response to ICIs in GC based on the TCGA-STAD dataset. Characterization of the TME was explored and further validated to reveal the underlying survival mechanisms and the potential therapeutic targets of GC. The GC patients were stratified into high- and low-risk groups based on the IRS. Patients in the high-risk group, associated with poorer outcomes, were characterized by significantly higher immune function. Further analysis showed higher T cell immune dysfunction and probability of potential immune escape. In vivo, we detected the expressions of *SERPINE1* by the quantitative real-time polymerase chain reaction (qPCR)in tumor tissues and adjacent normal tissues. In vitro, knockdown of *SERPINE1* significantly attenuated malignant biological behaviors of tumor cells in GC. Our signature can effectively predict the prognosis and response to immunotherapy in patients with GC.

## 1. Introduction

Gastric cancer (GC) is one of the most prevalent malignant tumors in humans and is the fourth leading cause of cancer-related deaths worldwide [1]. At present, the first-line regimen for advanced GC is based on platinum and fluorouracil with or without cetuximab, and the second-line regimen is based on paclitaxel with or without ramucirumab. Both regimens have been commonly used in clinical practice and have improved the prognosis of patients with advanced GC to some extent. However, the 5-year survival rate of these patients is still not satisfactory [2,3,4]. Recently, ICIs have emerged as a new treatment option for several malignancies, including advanced GC. Interim data for the KEYNOTE-811 study pre-liminary demonstrated the clinical efficacy and safety of using pembrolizumab (anti-PD-1 antibody) combined with trastuzumab and chemotherapy as the treatment for patients with HER-2-positive advanced GC or gastroesophageal junction adenocarcinoma [5]. The mid-term results showed that the overall response rate (ORR) and disease control rate (DCR) of the pembrolizumab group was significantly better than that of the placebo group. Besides, the ATTRACTION-2 study has evaluated the efficacy and safety of nivolumab (anti-PD-1 antibody) in treating patients with advanced GC in Asia after the failure of two or more lines of previous chemotherapy. The conclusion revealed a significant improvement of OS and PFS in the nivolumab group compared to the placebo group, which was found to be not likely affected by the level of PD-L1 expression [6].

Previous studies have found that only 10–26% of advanced gastric cancer could respond to immune checkpoint inhibitors [6,7,8]. Several biomarkers have been widely considered to be promising for predicting the immune response of gastric cancer, including PD-L1 combined positive score (CPS), microsatellite instability-high (MSI-H), tumor mutation load (TML), etc. However, according to the results of the ATTRACTION-2 study, the survival benefit of nivolumab in GC has nothing to do with the level of PD-L1 expression. Thus, only using the PD-L1 expression level as a screening criterion may ignore some immunotherapy responders. In addition, as another important indicator of immunotherapy response, MSI-H status was only identified in 22% of GC patients in the TCGA dataset [9]. These indicators share a common characteristic that they are merely based on the inherent characteristics of tumor cells, while ignoring the interaction between a variety of cells in the tumor immune microenvironment.

The tumor microenvironment (TME) of solid malignant tumors is extremely com-plex and heterogeneous, consisting of cancer cells, immune and stromal cells, cytokines, extracellular matrix (ECM), and vasculature. It could influence the growth of tumor cells and even their response to immunotherapy, which is mediated by tumor-associated fibroblasts, myeloid-derived suppressor cells, and stromal signal molecules such as transforming growth factor-β (TGF-β), CD8^+^ T cells, and NK cells in the immune microenvironment of tumor [10,11]. Therefore, it is important to have a systematic understanding of tumor cells, especially the immune microenvironment they reside in to further evaluate and screen potential immune checkpoint responders.

In this study, we established an immune risk signature (IRS) of GC with genes de-rived from three immune-related genes (IRGs) databases based on the TCGA-STAD cohort, which could predict the response to treatment and survival outcomes in GC patients treated with ICIs therapy. Next, we characterized the molecular and immune profile of the signature, and further analyzed the relationship between the signature and the TML. Finally, two independent immunotherapy cohorts of different tumor types were used to verify the stability and reliability of the signature.

## 2. Results

### 2.1. Construction of IRS

**Figure 1:**
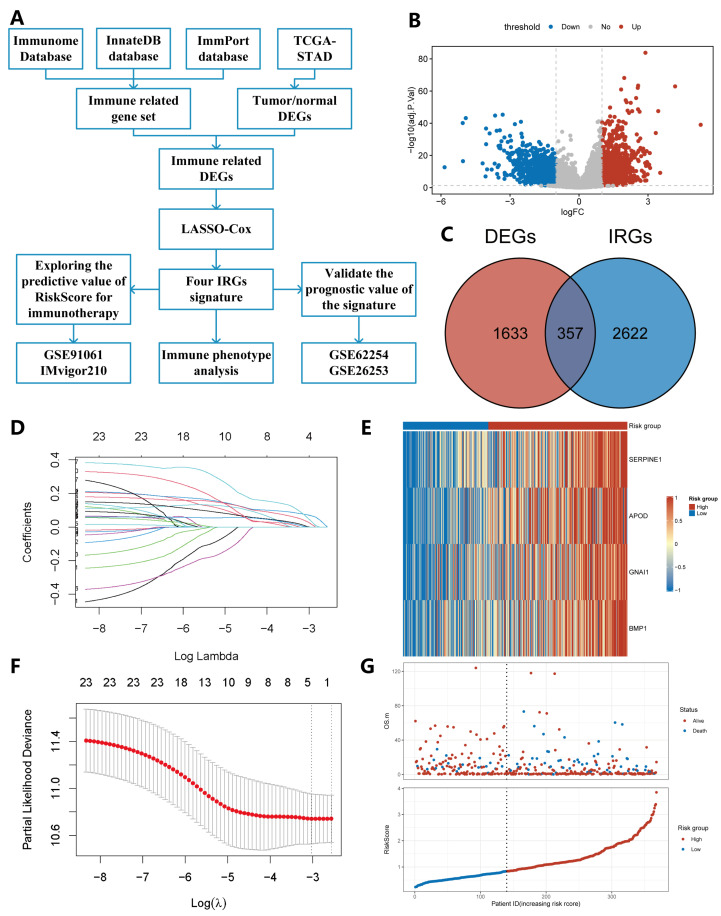
Flow chart of the study design and construction of the IRS. (**A**) Flow chart of our study. (**B**) DEGs in GC vs. adjacent normal tissues. (**C**) These DEGs were intersected with a combined IRGs set. (**D**) LASSO coefficient profiles of genes in TCGA-STAD. (**E**) Heatmap of the risk score consisting of four IRGs. (**F**) Selecting the best parameters by ten-fold cross-validation in the LASSO model. (**G**) Distribution of risk score, survival time, and status of patients in TCGA-STAD cohort.

We analyzed 375 gastric tumors and 32 normal tissue samples from GC patients available in the TCGA-STAD cohort and screened 1990 differentially expressed genes (DEGs), including 1093 down-regulated and 897 up-regulated genes (Figure 1B, Table S1). These DEGs were overlapped with 2979 IRGs using the Venn diagram, resulting in 357 immune-related DEGs being used for the subsequent regression analysis (Figure 1C, Table S2). A univariate Cox regression analysis indicated that 23 differentially expressed IRGs were significantly associated with OS (*p* < 0.05). Based on the 23 candidate genes above, we identified four IRGs (*SERPINE1*, *APOD*, *GNAI1*, *BMP1*) that could be enrolled in the construction of IRS via a LASSO-Cox regression analysis (Figure 1D,F, Table S3). An IRS based on the expression of identified candidate genes was calculated for each patient according to the following formula: risk score = *SERPINE1* × (0.1193) + *APOD* × (0.1189) + *GNAI1* × (0.1584) + *BMP1* × (0.2991). A survival analysis showed that a high expression of those four genes was associated with poor prognosis (Figure S1A–D).

After removing samples without complete clinicopathological characteristics from the TCGA-STAD cohort, a total of 368 GC patients were included in the subsequent analysis (Table S4). Based on the IRS, all patients were subdivided into high- (*n* = 228) and low-risk groups (*n* = 140) using the optimal cut-off value. The expression level of the four candidate genes and the overall survival (OS) with corresponding risk score were displayed in a heatmap and a scatterplot, respectively (Figure 1E,G).

### 2.2. The Prognostic Value of the IRS

**Figure 2:**
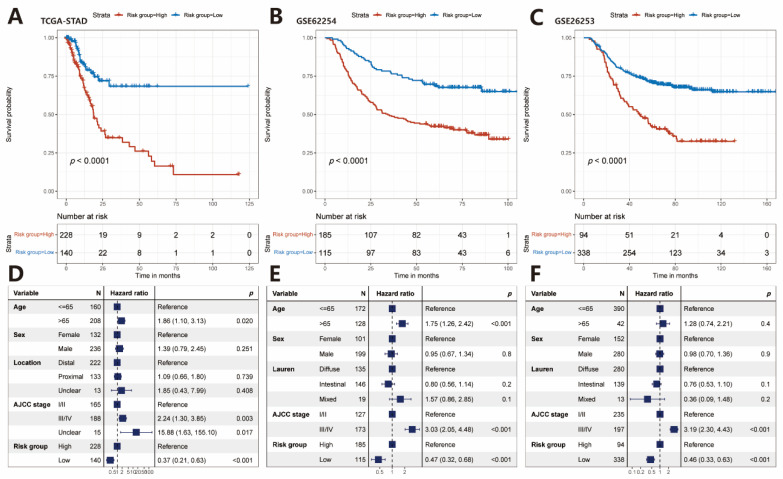
Relationship between risk score and patients’ survival in different cohorts of GC patients. K–M curves of OS according to risk groups in the TCGA-STAD discovery cohort (**A**), GSE62254 validation cohort (**B**), and GSE26253 validation cohort (**C**). Forest plot of multivariate regression analyses showing the association between risk score and patients’ survival in the three cohorts (**D**–**F**).

The Kaplan–Meier(K-M) survival analysis and log-rank test were employed to further identify the survival prediction power of the IRS in GC patients. The result showed that the OS of the high-risk group was much worse than that of the low-risk group (log-rank test, *p* < 0.0001; Figure 2A), indicating that a higher risk score could predict a worse prognosis. This association remained markedly significant in the multivariate Cox model after controlling several clinical features (HR, 0.37 [0.21–0.63], *p* < 0.001, Figure 2D). These clinical features were chosen because they have relatively complete data, and furthermore can affect the prognosis of cancer patients.

To further explore the prognostic value of the established IRS in different datasets, the K–M survival analysis and log-rank test were applied to the Gene Expression Omnibus (GEO) validation datasets. Heatmaps of the four identified IRGs expression levels and scatterplots of the OS with corresponding risk scores in two validation cohorts were shown in Figure S1E–H. As expected, patients with a low-risk score experienced significantly better OS compared with patients with a high-risk score (GSE62254: log-rank test, *p* < 0.0001, Figure 2B; GSE26253: *p* < 0.0001, Figure 2C). The multivariate Cox regression analysis further revealed that our immune risk signature could serve as an independent predictor of patients’ survival after being adjusted by clinicopathologic features including age, sex, Lauren classification, and American Joint Committee on Cancer (AJCC) stage in the two validation cohorts (GSE62254: HR, 0.47 [0.32–0.68], *p* < 0.001, Figure 2E; GSE26253: HR, 0.46 [0.33–0.63], *p* < 0.001, Figure 2F).

### 2.3. Estimation of TME Immune Infiltration and ICIs Response

**Figure 3:**
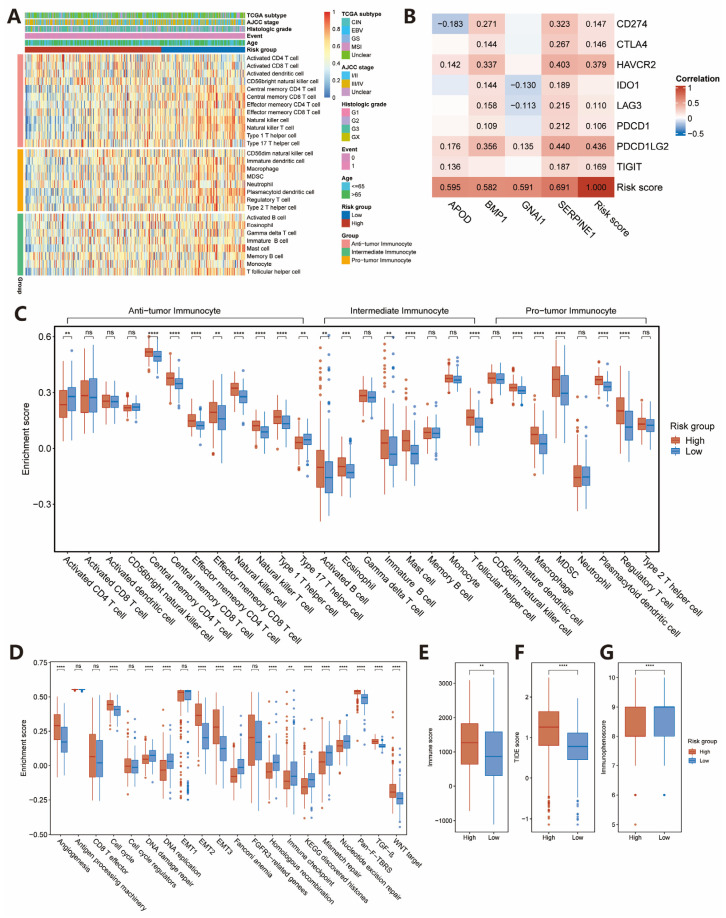
Transcriptome traits and clinical characteristics of TME phenotypes in the TCGA-STAD cohort. (**A**) ssGSEA identified the relative infiltration of 28 tumor-infiltrating immune cell types with different risk groups in the TCGA-STAD cohort. (**B**) Correlation matrix of risk score, four IRGs and seven immune-checkpoint-relevant genes (Spearman test, *p* < 0.05). (**C**,**D**) The relationship between risk score and 28 tumor-infiltrating immune cell types or pathways using ssGSEA. (**E**–**G**) Comparison of Immune, TIDE scores, and IPS between high- and low-risk groups in TCGA-STAD cohort. Wilcoxon test, ** *p* < 0.01; *** *p* < 0.001; **** *p* < 0.0001; ns, not statistically significant.

Considering that the genes included in the IRS were extracted from the IRGs database, we speculated that they might regulate the immune cells infiltration. A single sample gene set enrichment analysis (ssGSEA) was applied to identify the immune cell infiltration patterns and to calculate the normalized enrichment scores of 28 immune cell subpopulations (Figure 3A,C). The result demonstrated that anti-tumor lymphocyte cell subpopulations were enriched in the high-risk group, including activated CD4^+^ T cell, central memory CD4^+^ T cell, central memory CD8^+^ T cell, effector memory CD4 T cell, effector memory CD8 T cell, natural killer cell, natural killer T cell, and type 1 T helper cell. In addition, pro-tumor lymphocyte cell subpopulations, such as immature dendritic cell, macrophage, myeloid-derived suppressor cell (MDSC), plasmacytoid dendritic cell and regulatory T cell were also significantly enriched in the high-risk group. Similar results were found for the GEO validation datasets (GSE62254: Figure S2A,C; GSE26253: Figure S2B,D).

Ten kinds of pathways were quantified by using ssGSEA as well. These analyses confirmed that the high-risk group was significantly associated with epithelial to mesenchymal transition (EMT), pan-fibroblast TGF-β response signature (Pan-F-TBRS), TGF-β, WNT target, and angiogenesis pathways, whereas the low-risk group was associated with DNA damage repair, immune checkpoint, mismatch repair, and nucleotide excision repair pathways (Figure 3D). Figure 3B shows a positive correlation between expression of most of the four IRGs and immune checkpoint-relevant genes. We also found the risk score was significantly and positively correlated to immune checkpoint-relevant genes and IRGs.

To further explore the fractions of stromal and immune cells in GC tissues, estimation of stromal and immune cells in malignant tumor tissues using expression data (ESTIMATE) algorithm was applied to calculate the immune, ESTIMATE, stromal scores, and tumor purity. The results illustrated that patients in the high-risk group had significantly higher immune, ESTIMATE, stromal scores, but lower tumor purity compared with those in the high-risk group (Figure 3E and Figure S3A–C). In addition, the cytolytic activity score, an assessment of anti-tumor immune activity, was also higher in the high-risk group (Figure S3D).

To the best of our knowledge, a patient’s response to immunotherapy can be inferred by the immunophenoscore (IPS) and tumor immune dysfunction and exclusion (TIDE) score [12,13]. Our result revealed that patients in the high-risk group displayed higher TIDE scores and lower IPS than those in the low-risk group (Figure 3F,G). These data also suggested that patients in the low-risk group might be more sensitive to immunotherapy compared with those in the high-risk group. In addition, the high-risk group showed significantly higher T cell dysfunction and T cell exclusion scores. (Figure S3E,F). Previous studies have demonstrated the important role of cancer-associated fibroblasts (CAFs) in shaping the immunosuppressive TME by regulating tumor-associated myeloid cells (TAMs) to induce a pro-tumor phenotype [14]. Thus, we calculated CAF scores in this study and found it significantly higher in the high-risk group (Figure S3G). Moreover, the low-risk group was found to exhibit higher MSI (Figure S3H). We also detected the relative expression levels of marker genes of CAFs (*MMP2*, *ACTA2*, *TAGLN*, *THY1*, *TNC*, *PDPN*, *PDGFRA*, *PDGFRB*, *GLI1*, *CXCL12*, *GREM1,* and *VIM*) [15,16] and T cell exhaustion (*LAG3*, *CTLA4*, *PD1*, *PD-L1*, and *HAVCR2*) [17], which were consistent with the results calculated by the TIDE algorithm (Figure S3I,J).

We further validated TIDE scores and IPS in the GSE62254 and GSE26253 cohorts. Consistently, our analysis also revealed that the TIDE score was significantly elevated in the high-risk group (Figure S3L,M), and IPS was significantly decreased in the high-risk group, but the difference of IPS in the GSE62254 cohort was not significantly different (Figure S3N,O). These results together indicated that GC patients with lower risk scores were more sensitive to immunotherapy.

### 2.4. Somatic Variations in Two Subgroups

**Figure 4:**
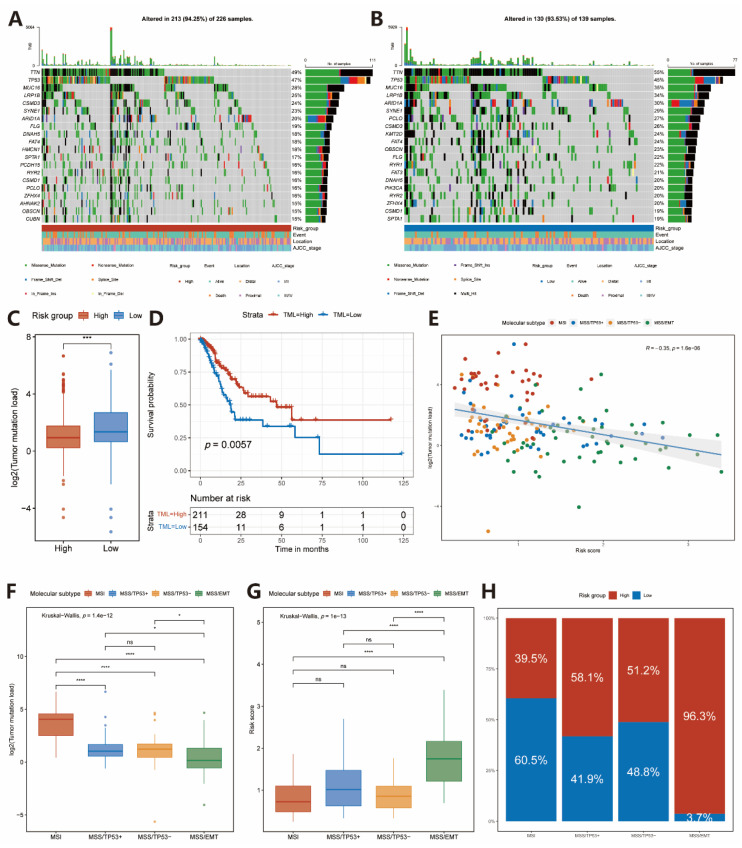
Comparison of tumor mutations between high- and low-risk groups. (**A**,**B**) The oncoPrint of high- and low-risk groups in the TCGA-STAD cohort. (**C**) TML difference in the high- and low-risk groups. Wilcoxon test, *** *p* < 0.001. (**D**) Kaplan–Meier curves for high- and low-TML groups of the TCGA-STAD cohort. Log-rank test, *p* = 0.011. (**E**) The relationship between risk score and TML in TCGA-STAD cohort (Spearman test, *p* < 0.0001). The dotted color indicates the ACRG molecular subtypes of GC. (**F**) TML difference in different ACRG molecular subtypes of the TCGA-STAD cohort. Steel–Dwass test, * *p* < 0.05; *** *p* < 0.001; ns, not statistically significant. (**G**,**H**) Distribution of the risk score and percentage of the high-risk group in different ACRG molecular subtypes of the TCGA-STAD cohort. Steel–Dwass test, **** *p* < 0.0001; ns, not statistically significant.

Among the 368 enrolled TCGA-STAD patients, 365 patients had available somatic mutation data, and three patients without somatic mutation data were excluded from the subsequent analysis. Next, a differential somatic mutation analysis was performed between high- and low-risk groups in the TCGA-STAD cohort. In the top 20 genes, the highest mutation frequency, all of them harbored higher mutation frequency in the low-risk group than in the high-risk group except for *TP53* (Figure 4A,B).

A lot of evidence has demonstrated that higher TML is correlated with an improved response to ICIs therapy and prolonged OS [18,19]. Thus, we compared the TML between high- and low-risk groups and found patients in the low-risk group behaving a significantly higher TML than those in the high-risk group (Figure 4C). Moreover, patients with high TML had a significantly better OS compared to patients with low TML (Figure 4D).

In 2015, Cristescu et al. classified GC into four molecular subtypes based on the integrated genetic characteristics: MSI, MSS/EMT, MSS/TP53^+^ and MSS/TP53. A significant negative correlation was observed between TML and risk score (Spearman coefficient: R = −0.35, *p* < 0.0001, Figure 4E). Of the four subtypes, MSI showed the best overall prognosis, followed by MSS/TP53^+^, MSS/TP53^−^, and MSS/EMT [20]. We assessed the relationship between four molecular subtypes and TML in the TCGA-STAD cohort, and we found that the MSI subtype was associated with the highest TML, while the MSS/EMT subtype demonstrates the lowest TML of the four subtypes (Figure 4F). We also explored the distributions of risk scores in the four subtypes of GC from TCGA-STAD, GSE62254, and GSE26253 cohorts, respectively. As a consequence, most patients with high-risk scores were located in the MSS/EMT subtype group (TCGA-STAD: Figure 4G,H; GSE62254: Figure S4A,C; GSE26253: Figure S4B,D).

### 2.5. Immunotherapeutic Benefits Predicted by the IRS

**Figure 5:**
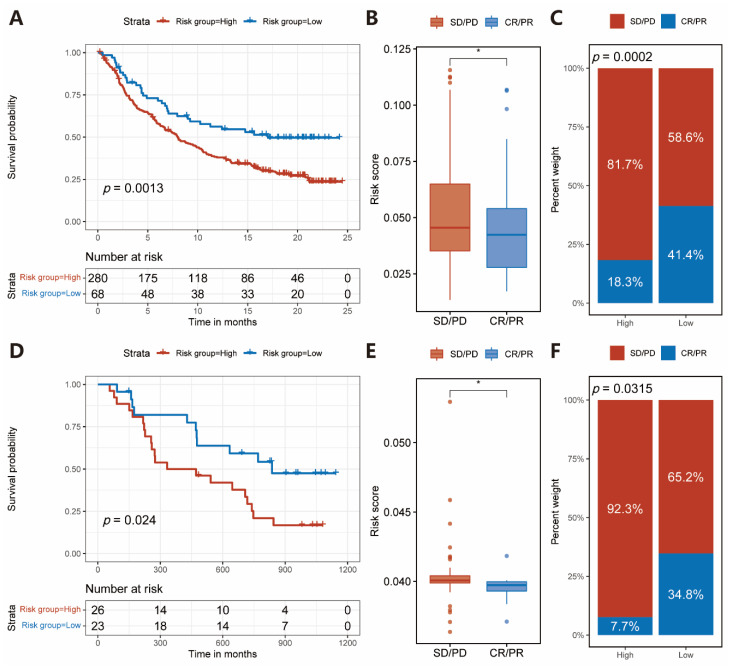
Response to immunotherapy in patients of urothelial cancer and melanoma with different risk groups divided by risk score. (**A**) K–M curve for patients of urothelial cancer in the high- and low-risk groups in the IMvigor210 cohort. Log-rank test, *p* = 0.0013. (**B**) Risk score in groups with a different clinical response to immunotherapy in the IMvigor210 cohort. Wilcoxon test, * *p* < 0.05. (**C**) The proportion of patients with a response to immunotherapy in high- and low-risk groups in the IMvigor210 cohort. Pearson’s Chi-squared test, *p* = 0.0002. (**D**) K–M curve for patients of melanoma in the high- and low-risk groups in the GSE91061 cohort (Log-rank test, *p* = 0.024). (**E**) Risk score in groups with a different clinical response to immunotherapy in the GSE91061 cohort. Wilcoxon test, * *p* < 0.05. (**F**) The proportion of patients with response to ICIs therapy in high- and low-risk groups in the GSE91061 cohort. Fisher’s exact test, *p* = 0.0315.

To evaluate the potential application value of IRS in predicting patients’ response to ICIs therapy, we further analyzed our IRS in two cohorts of urothelial cancer (IMvigor210) and melanoma (GSE91061) with integrated clinical information of immunotherapy. Patients in the two cohorts were stratified into high- or low-risk groups based on the IRS, respectively. K-M survival analysis showed that patients in the low-risk group had a much better prognosis than those in the high-risk group from the IMvigor210 cohort (log-rank test, *p* = 0.0013, Figure 5A). Patients with complete or partial response disease (CR/PR) tended to have a decreased risk score compared with those with stable or progressive disease (SD/PD) in the IMvigor210 cohort (Wilcoxon test, *p* = 0.026, Figure 5B). We also found that patients in the low-risk group had a better response to immunotherapy than patients in the high-risk group (Pearson’s Chi-squared test, *p* = 0.0002, Figure 5C) in the IMvigor210 cohort. A similar outcome was observed in the GSE91061 cohort (log-rank test, *p* = 0.024, Figure 5D; Wilcoxon test, *p* = 0.042, Figure 5E; Fisher’s exact test, *p* = 0.0315, Figure 5F). Moreover, in the IMvigor210 cohort, low-risk group patients possessed higher TML and neoantigen burden (Figure S5A,B). Taken together, our results suggested that IRS could be applied to predict patients’ response to immunotherapy and their prognosis both in urothelial cancer and melanoma.

### 2.6. Construction and Verification of Nomogram

**Figure 6:**
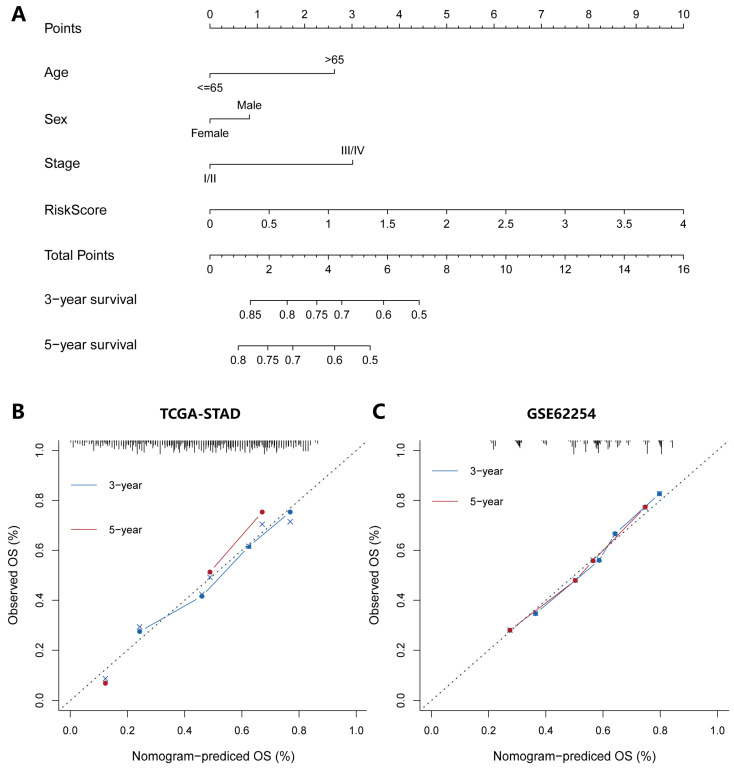
Construction and evaluation of a nomogram based on the IRS to predict the 3-year and 5-year OS for patients with gastric cancer. (**A**) Nomogram was constructed with the TCGA-STAD cohort for predicting the probability of 3-year and 5-year OS for GC patients. (**B**) Calibration plot for 3-year and 5-year OS in the TCGA-STAD cohort. (**C**) Calibration plot for 3-year and 5-year OS in the GSE62254 cohort.

We built a nomogram combining risk score and independent clinical prognostic factors including age, sex, and AJCC stage, to predict the probability of 3-year and 5-year OS in the TCGA-STAD cohort (Figure 6A). The calibration curve illustrated that the predictions and actual observations matched well (Figure 6B). Furthermore, the nomogram was validated in the GSE62254 and GSE26253 gastric cancer datasets, and the 3-year and 5-year calibration curves are respectively shown in Figure 6C and Figure S5C.

We further compared the area under the curve analyses (AUC) for the risk score, TML, PD-L1 expression level, TIDE score, and IPS, we found the risk score to have the best ability of predicting prognosis in GC patients (Figure S5D).

### 2.7. Validation of the IRGs in Clinical Tissue Samples

**Figure 7:**
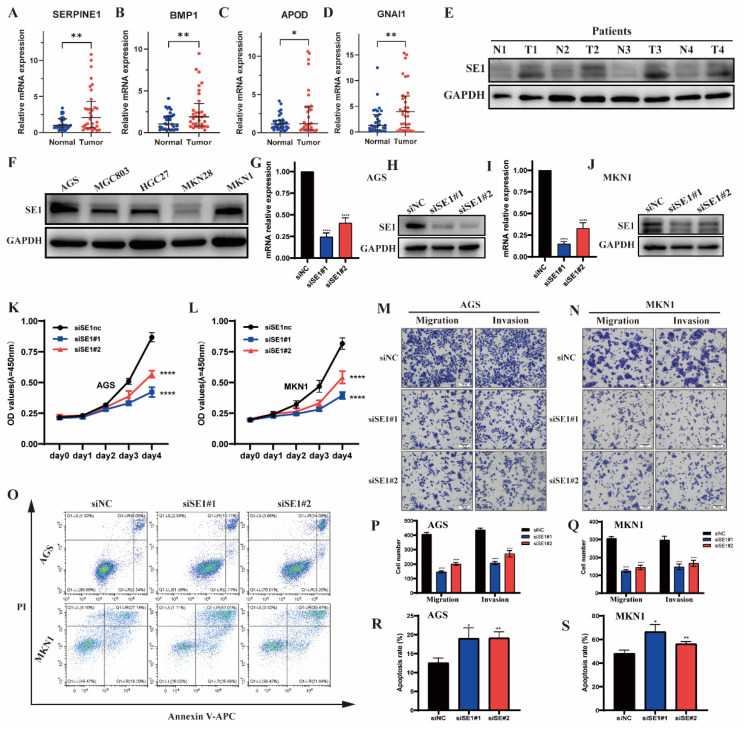
*SERPINE1* promotes the malignant biological behaviors of GC cells. *SERPINE1* (**A**), *APOD* (**B**), *GANI1* (**C**) and *BMP1* (**D**) mRNA and SERPINE1 (**E**) protein levels were measured in GC tissues, and adjacent normal tissues were paired by qPCR and Western blotting, respectively. Western blotting analyses of SERPINE1 protein levels in GC cell lines (**F**). Western blot and qPCR analyses of SERPINE1 levels in GC cell lines transfected with the si*SERPINE1* (**G**–**J**). CCK8 assay of cell growth with *SERPINE1* silencing and control group (**K**,**L**). Representative images and quantification of migration and invasion in *SERPINE1* silencing and control GC cell lines (**M**,**N**,**P**,**Q**). Apoptosis assay and the quantitative analysis of *SERPINE1* in silencing and control group (**O**,**R**,**S**). Data were presented as the mean ± SD. Wilcoxon test, * *p* < 0.05; ** *p* < 0.01; **** *p* < 0.0001.

To further confirm the reliability of the identified IRGs, we used qPCR to compare the mRNA expression of 32 pairs of matched GC and adjacent gastric tissue samples. The protein expression of *SERPINE1* in four paired GC and adjacent gastric tissues was measured by Western blotting. The results showed that all four mRNA were significantly overexpressed in GC tissues compared with adjacent normal tissues (Figure 7A–D). Similar results were obtained in Western blotting analysis, in which *SERPINE1* was significantly overexpressed in GC tissue (Figure 7E).

### 2.8. Silencing of SERPINE1 Inhibited the Proliferation, Invasion, Metastasis but Promoted the Apoptosis of GC Cells

SERPINE1 protein levels in five human GC cell lines were measured by Western blotting analyses as demonstrated in Figure 7F. We chose two cell lines (AGS, MKN1) which express high amounts of SERPINE1. Next, these cells were transfected with siRNA, and the silencing effect of siRNA was validated by qPCR and Western blotting analyses (Figure 7G–J). As shown in Figure 7K–S, silencing of *SERPINE1* resulted in inhibited growth, migration, and invasion capacities in both AGS and MKN1 GC cell lines. Moreover, *SERPINE1* silencing significantly increases apoptosis in both AGS and MKN1 cells.

## 3. Discussion

ICIs therapy has proven its high suppression efficacy in tumor initiation and development in patients with advanced GC [6,7]. Only a handful of GC patients could truly benefit from ICIs therapy due to the high heterogeneity. Hence, the identification of potential responders to ICIs before treatment initiation could be reasonable. However, accurately predicting the response to ICIs therapy has never been an easy task to achieve. Previous studies have shown that TME plays an important role in both tumor progression and patient response to immunotherapy [21,22]. Therefore, a deeper understanding of TME immune infiltration can provide us with a very powerful tool to identify patients responsive to GC ICIs therapy. In this study, by using the transcriptome profile of the TCGA-STAD cohort, a robust prognostic IRS based on four IRGs was developed, and its efficacy was further verified in four independent validation cohorts including two immunotherapy cohorts. Therefore, the IRS could be possible to characterize the TME cell infiltration patterns and serve as a potential biomarker for predicting the prognosis and responsiveness to ICIs therapy in GC patients.

The four genes (*SERPINE1*, *APOD*, *GNAI1*, *BMP1*) involved in the IRS have been previously reported to be associated with the prognosis of GC. *SERPINE1* (Serpin peptidase inhibitor, clade E, member 1) is the main regulator of the plasminogen activator (PA) system which relates to the tumor growth, invasion, and metastasis via the activation of matrix metalloproteinases (MMPs) as well as latent growth factors [23]. High *SERPINE1* expression has been observed in several tumor types and has been described as a poor prognostic marker [23,24,25]. Next, we silenced *SERPINE1* to further investigate their effects on AGS and MKN1 cells in vitro. The results demonstrated that silencing *SERPINE1* significantly inhibited the proliferation, invasion, and metastasis, but promoted the apoptosis of GC cells. *APOD*, which encodes a component of high-density lipoprotein has been reported to promote cell migration through interaction with growth factors [26]. A higher *APOD* expression level detected in breast and colorectal cancer tissues has been proven to be associated with a poor prognosis [27,28]. *BMP1* (bone morphogenetic protein 1) is a member of the astacin superfamily, whose main function is to promote the formation and development of the extracellular matrix [29]. It is well known that the extracellular matrix acts as a repository of TGF-β whose release relies heavily on *BMP1* [30]. Therefore, activation of the TGF-β signaling pathway requires BMP1 [31]. In GC, *BMP1* can promote the development of cell growth and metastasis through activation of the TGF-β signaling pathway [32], consistent with our previous results in signal pathway analysis via ssGSEA. *GNAI1* (G protein subunit alpha i1) is a member of the Gαi family and functions to suppress adenylate cyclase activity. To date, only a handful of studies have shown that the high expression and low DNA hypermethylation of *GNAI1* were significantly associated with poor prognosis in GC [33].

In this study, preliminary findings of phenotype analysis confirmed a better immune status of GC patients in the high-risk group from the TCGA-STAD cohort. This interesting phenomenon is yet to be explained in previous studies on immune-related genes of GC [34,35,36]. We speculated that an inhibitory program might facilitate cancer cells escaping from host immune surveillance. Tumor cells with high TML tend to have a relatively high level of tumor antigen. Our study shows that the high-risk group with a higher TML is considered to help the immune system recognize the tumors and stimulate the proliferation and anti-tumor response of anti-tumor immune cells. However, when exposed to tumor antigens for a long time, the expression of tumor suppressors such as *PD-1*, *CTLA4*, *TIGIT*, *TRIM3*, and *LAG3* in the microenvironment will be significantly upregulated, which will lead to impaired killing function and exhaustion of CD8+ T cells [37,38]. Thus, although the high-risk group has a higher immune cell infiltration, the CD8+ T in the TME lost their original anti-cancer functions. Our later analysis also finds that, firstly, the degree of tumor-promoting immune cell infiltration in the high-risk group is significantly higher than that in the low-risk group; secondly, the TIDE algorithm shows the dysfunction and exhaustion of T cells in the high-risk group; and finally, some suppressors in the TME, such as *PD-1*, *CTLA4*, *TIGIT*, *TRIM3*, *LAG3*, CAFs marker genes are also significantly higher in the high-risk group. We also found that the high-risk group was associated with the immunosuppressive pathways, such as TGF-β and WNT signaling pathways, which were significantly enriched. Activation of TGF-β and EMT pathways, as well as CAFs proliferation, can inhibit T cell-mediated tumor killing and reduce the transport of T cells to the tumor [39,40]. Preclinical evidence demonstrated that TGF-β blockade combined with PD-L1 antibody can decrease TGF-β signal transduction in stromal cells, promote T cell infiltration to tumor center, induce the anti-tumor immune effect, and bring a tumor regression effect [41,42,43]. As a novel target, TGF-β opens a new chapter in tumor immunotherapy.

According to our analysis, we found a negative correlation between the risk score and TML in the TCGA-STAD cohort. Among the four Asian Cancer Research Group (ACRG) GC subtypes, subtype MSS/EMT got the highest risk scores and lowest TML, and it also had the highest proportion of patients with high-risk scores. These findings are consistent with the fact that stromal activation is the vital mechanism of resistance to ICIs therapy [39,40]. In GC, patients with MSI demonstrate a higher sensitivity to ICIs therapy and a more favorable prognosis compared with patients with MSS [44]. Our data indicated that patients with MSI subtype showed a lower risk score and the highest TML among molecular subtypes, suggesting that the IRS may be useful for predicting clinical benefits in GC patients treated with immunotherapy.

Nevertheless, there were several limitations in our work. Firstly, because the datasets of GC patients used in this study were obtained from different public databases, certain heterogeneity may exist in our work. Secondly, due to a lack of GC immunotherapy cohorts measured at the transcriptome level, we had to perform a model validated by using urothelial cancer and melanoma datasets. Finally, this study was restricted to in vitro experiments, lacking the validation of in vivo experiments, which is a limitation of our study. More in vivo experiments should be done to further confirm our findings in the future.

## 4. Materials and Methods

### 4.1. Study Design and Data Collection

A flow chart of the study design is shown in Figure 1A. We systematically searched for GC gene-expression datasets that were publicly available and reported integral clinical annotations. RNA-seq data, somatic mutation data (SNPs and small INDELs, MuTect2 Variant Aggregation and Masking) and clinical features of the TCGA-STAD cohort were downloaded from the Genome Data Commons Data Portal (GDC) (https://portal.gdc.cancer.gov, accessed date: 20 March 2021). Microarray or RNA-seq data for validation was downloaded from GEO (https://www.ncbi.nlm.nih.gov/geo/query/acc.cgi, accessed date: 20 March 2021). We only retained GC patients that met the following criteria: (1) Have mRNA expression data; (2) Have both recurrent status and OS information; (3) Have clinical features, such as AJCC stage II/III, age, sex, tumor location, Lauren classification. Patients without survival information were excluded from further studies. Therefore, three datasets with a total of 1100 GC patients were enrolled for analysis, including TCGA-STAD (*n* = 368), GSE62254 (*n* = 300), GSE26253 (*n* = 432) [45]. Due to the clinical information in TCGA-STAD cohort with complete clinical information, it was employed as a discovery cohort to construct IRS. The remaining two datasets from GEO were used as validation sets to test the predictive ability of the signature. The raw gene expression matrix is available as supplementary file S1.

The IRGs lists were downloaded from Immunome Database (https://www.innatedb.com/browse.jsp, accessed date: 20 March 2021), InnateDB database (https://www.innatedb.com/browse.jsp, accessed date: 20 March 2021), and ImmPort database (https://www.immport.org/shared/genelists, accessed date: 20 March 2021). After removing duplicate genes, a combined gene set that included 2979 unique genes was used for further analysis.

### 4.2. Establishment and Validation of IRS

To establish the IRS, we conducted a comprehensive analysis. First, gene expression level inferred by FPKM (Fragment Per Kilobase Millon) in the TCGA-STAD cohort was transformed into TPM (Transcripts Per Kilobase Millon). The differences in mRNAs expression between GC tissues and normal tissues were screened by R package “limma” (version 3.48.0). Genes with adjusted *p*-value < 0.05 and |log_2_ fold change| > 1 were classified as DEGs. The intersection of the combined IRGs and DEGs was selected as the set of the differentially expressed IRGs for a subsequent regression analysis.

Next, univariate Cox regression was performed to identify genes significantly correlated with OS (*p* < 0.05). LASSO-Cox with 10-fold cross validation (R package “glmnet”, version 4.1-1) was used to identify novel IRGs and construct the signature. Afterwards, the risk score based on the model was calculated by the following formula: risk score = $\sum_{i=1}^{n}{\mathrm{coef}}_{i}{*\mathrm{Exp}}_{i}$, where *n* is the total number of genes included in the final signature, *i* indicates the *i*th gene in the signature, and ${\mathrm{coef}}_{i}$ and ${\mathrm{Exp}}_{i}$ represent the coefficient and the expression level of the *i*th gene, respectively. High- and low-risk groups were determined based on the best cut-off point obtained from the R package “survminer” (version 0.4.9). Eventually, the survival analysis for different groups was performed using the K–M method and log-rank test with R package “survival” (version 3.2-11) and “survminer” (version 0.4.9).

### 4.3. Estimation of TME Immune Infiltration and Functional Annotation

To quantify the immune infiltration for each sample, ssGSEA was applied based on 28 immune cell gene sets and 10 immune-related pathways collected from the previous study [12,39]. The enrichment scores calculated by the ssGSEA algorithm indicated the relative abundance of each type of immune cell or pathway. The ssGSEA score was normalized to unify distribution from 0 to 1. Furthermore, the Wilcoxon test was carried out to quantify the difference in the enrichment level of immune cells and pathways between the high- and low-risk groups. The ssGSEA analysis was performed using R package GSVA (version 1.40.1), and the boxplots were conducted using R package “ggpubr” (version 0.4.0).

The ESTIMATE algorithm was carried out to quantify the immune and stromal components in TME by utilizing the ‘estimate’ R package, which was employed to calculate the immune, stromal, tumor purity, and estimate scores [46].

### 4.4. Quantification of the Immunotherapy Response

The potential response of patients to immunotherapy was inferred by IPS as well as TIDE score. Generally, a higher IPS and lower TIDE score may indicate a better response to immunotherapy [12]. The TIDE score can be obtained by the TIDE algorithm and calculated online (https://tide.dfci.harvard.edu/, accessed date: 17 August 2021) [13]. In addition, MSI, Dysfunction, Exclusion and CAFs scores can also be obtained by TIDE algorithm.

IPS of each TCGA-STAD sample can be downloaded from https://tcia.at/patients (accessed date: 17 August 2021). The IPS was constructed as proposed by Charoentong et al. [12], and a respective R code used to calculate the IPS of GEO validation sets was downloaded from https://github.com/MayerC-imed/Immunophenogram (accessed date: 17 August 2021). The cytolytic activity was calculated through a geometric mean expression of six genes, five granzymes (*GZMA*, *GZMB*, *GZMH*, *GZMK*, *GZMM*), and one perforin (*PRF1*), to quantify the level of cytotoxic immune cell activity [47].

### 4.5. Correlation between the IRS and Somatic Variants

A myriad of evidence has demonstrated that TML is also associated with anti-tumor immunity. TML presence can increase T cell infiltration and trigger the T-cell response in tumor tissue [47,48]. Therefore, we analyzed the somatic mutation data from TCGA-STAD cohort to explore the difference in genomic alterations between two groups. We first compared the TML of the high-risk group with that of the low-risk group. Next, patients were divided into high- and low-TML groups based on the cut-off point of TML. Furthermore, a K–M survival analysis was performed to confirm the relationship between TML and prognosis.

In 2015, The ACRG described four molecular subtypes based on gene the expression data of GC [20]; we compared the TML of these four subtypes and explored the relationship between risk scores and TML in the TCGA-STAD cohort.

### 4.6. IRS Predicting ICIs Therapy Benefits

Two independent immunotherapeutic cohorts, IMvigor210 and GSE91061 of advanced urothelial cancer and melanoma, were downloaded and analyzed to validate the prediction value for immunotherapy of the signature. Detailed clinical features and complete gene expression profiles of the IMvigor210 cohort were integrated into an R package, which could be extracted freely from http://research-pub.gene.com/IMvigor210CoreBiologies (accessed date: 3 July 2021) under the Creative Commons 3.0 license. After screening, a total of 298 urothelial cancer and 49 melanoma patients who received immunotherapy with complete clinical information were analyzed to calculate the risk scores.

### 4.7. Construction of Nomogram

Based on a multivariate Cox analysis, the risk score and clinical features including age, sex, and AJCC stage were together used to construct the nomogram by the “rms” (version 6.2-0) and “survival” packages in R to predict the probability of a 3-year and 5-year OS. The calibration plots were drawn to assess the consistency between actual and predicted survival both in discovery and validation cohorts.

Efficiency of the parameters, including risk score, TML, PD-L1 expression level, TIDE score, IPS, were assessed through the receiver operating characteristic (ROC) curve and AUC.

### 4.8. Cell Lines and Cell Culture

The human GC cell lines AGS, MKN-1 were obtained from the Shanghai Institute of Cell Biology, Chinese Academy of Sciences (Shanghai, China). AGS was grown in DMEM/F12 (Gibco, Waltham, MA, USA). MKN-1 was maintained in an RPMI-1640 medium (Gibco, Waltham, MA, USA). All culture media were supplemented with 10% fetal bovine serum (FBS, Gibco, Waltham, MA, USA) and 1% penicillin/streptomycin (Gibco, Waltham, MA, USA). All cells were cultured in a 37 °C humidified incubator with 5% CO_2_.

### 4.9. Small Interfering (si)RNA Transfection

All human GC cell lines were seeded in 6 well plates and transfected with 50 nM si*SERPINE1* or siNC using Lipofectamine 3000^®^ (Invitrogen; Thermo Fisher Scientific, Waltham, MA, USA) according to the manufacturer’s protocol. The transfected cells were incubated at 37 °C for 48 h to 72 h. The transfection efficiency was determined by Western blotting and qPCR. siRNAs used in this study are provided in Table S5.

### 4.10. Quantitative Real-Time Polymerase Chain Reaction (qPCR)

RNA extraction from cells and GC tissue was conducted using TRIzol reagent (Takara, Kusatsu, Japan) following the manufacturer’s protocol. Subsequently, mRNA was transcribed to cDNA using the 5× Master Mix and qRT-PCR was performed using the 2× SYBR^®^ Green I (Accurate Biotechnology, Changsha, Hunan, China) with gene-specific primers on an ABI QuantStudio (Applied Biosystems; Thermo Fisher Scientific, Waltham, MA, USA). Details of qPCR primers used are listed in Table S6.

### 4.11. Western Blot Analysis

Total proteins extracted from cells and GC tissue lysates were separated by 10% SDS-PAGE and transferred onto 0.45 um polyvinylidene difluoride (PVDF) membranes (Merck Millipore; Darmstadt, Germany). After blocking with 5% non-fat milk, the membranes were incubated with the primary antibodies (anti-SERPINE1[13801-1-AP], anti-GAPDH [60004-1-Ig], Proteintech, Wuhan, China) overnight at 4 °C, followed by incubation with a horseradish peroxidase-conjugated secondary antibody for 1 h at room temperature. The immunoreactive bands were detected by enhanced chemiluminescence reagents (Merck Millipore; Darmstadt, Germany).

### 4.12. Cell Proliferation, Migration, Invasion, and Cell Apoptosis Assays

Cells were harvested at 48 h after transfection and were seeded into 96-well plates at 1000 cells/well and grown for 0–4 days at 37 °C in a humidified incubator with 5% CO_2_. Then, 10% CCK-8 reagents were added to each well, and the cells were further incubated for two hours. The optical density at 450 nm was measured using a microplate reader.

Assays for cell migration and invasion, 5 × 10^4^ cells/well in 300 μL media without fetal bovine serum were plated on the top chambers of a transwell insert (Corning Costar, Cambridge, MA, USA) with or without Matrigel coating (Corning Costar, Cambridge, MA, USA), and added media supplemented with 10% fetal bovine serum was applied to the lower chambers. After 18–26 h, the migrated or invaded cells were fixed with 4% polyoxymethylene, stained with 0.1% crystal violet and counted under an inverted microscope.

Cell apoptosis assay was performed using Annexin V-APC Apoptosis Detection Kit (KeyGEN, Nanjing, Jiangsu, China) following the manufacturer’s instruction. The percentage of positive cells was detected using CytoFLEX (Beckman Coulter, Brea, CA, USA). CytExpert (Version 2.4) was used for analysis.

### 4.13. Statistical Analyses

All statistical analyses were conducted using R version 4.1.0 software (https://cran.r-project.org/) (accessed date: 17 June 2021) and GraphPad Prism was also applied for the data analysis. Wilcoxon rank-sum test with false discovery rate (FDR) correction was used to compare quantitative variables between two groups, and the Kruskal–Wallis test, followed by the post-hoc Steel–Dwass test, was used for multiple comparisons. Chi-square test was employed for comparisons of qualitative variables. Survival curves were plotted using K–M plotter and compared by a log-rank test. Univariate and multivariate Cox regression analyses were conducted to determine factors with independent prognostic value. The AUC was quantified using the “timeROC” R package (version 0.4). All statistical *p* values were two-sided, with *p* < 0.05 as statistically significant.

## 5. Conclusions

Based on the immune-related DEGs between GC and adjacent normal tissues, an immune risk signature was established for GC patients treated with ICIs therapy. Our in vivo experiment strengthened our finding that silencing the main gene *SERPINE1* could significantly inhibit the malignant biological behavior of GC cells. After calculating the risk scores, GC patients assigned to the high-risk group tended to have better a response to ICIs therapy. In further analysis of TME, the infiltration of immunosuppressive factors and the loss of T cell effector function were found to be related to poor prognosis of GC patients in the high-risk group. Taken together, these results put forward a special view on the TME of GC, and our IRS showed a potential clinical applicability to discriminate those GC patients who might benefit from ICIs therapy.

## Data Availability

Data is contained within the article and supplementary material.

## Supplementary Materials

The following supporting information can be downloaded at: https://www.mdpi.com/article/10.3390/ph15111401/s1, Figure S1. Construction of the IRS. Figure S2. Transcriptome traits and clinical characteristics of TME phenotypes in the GEO validation cohorts. Figure S3. Biological function analysis between high- and low-risk groups in the three cohorts. Figure S4. Relationship between risk score and ACRG molecular subtypes in the GEO validation cohorts. Figure S5. The predictive value of risk score was validated in the testing datasets. Table S1. Differentially expressed genes. Table S2. Immune-related DEGs. Table S3. Results of univariate regression and multivariate regression analysis of the four included genes. Table S4. Clinical characteristics of patients with gastric cancer in 3 datasets. Table S5. *SERPINE1* siRNAs sequences. Table S6. The primer sequences of 4 included genes.
