# Supplementary material for: A SERPINE1-Based Immune Gene Signature Predicts Prognosis and Immunotherapy Response in Gastric Cancer
Source: Pharmaceuticals (Basel). 2022 Nov 14;15(11):1401. doi: 10.3390/ph15111401 (PMC9692477; doi:10.3390/ph15111401)
Supplement: Supplementary file 1 [file pharmaceuticals-15-01401-s001.zip › suplementary.pdf]

Supplementary Figures

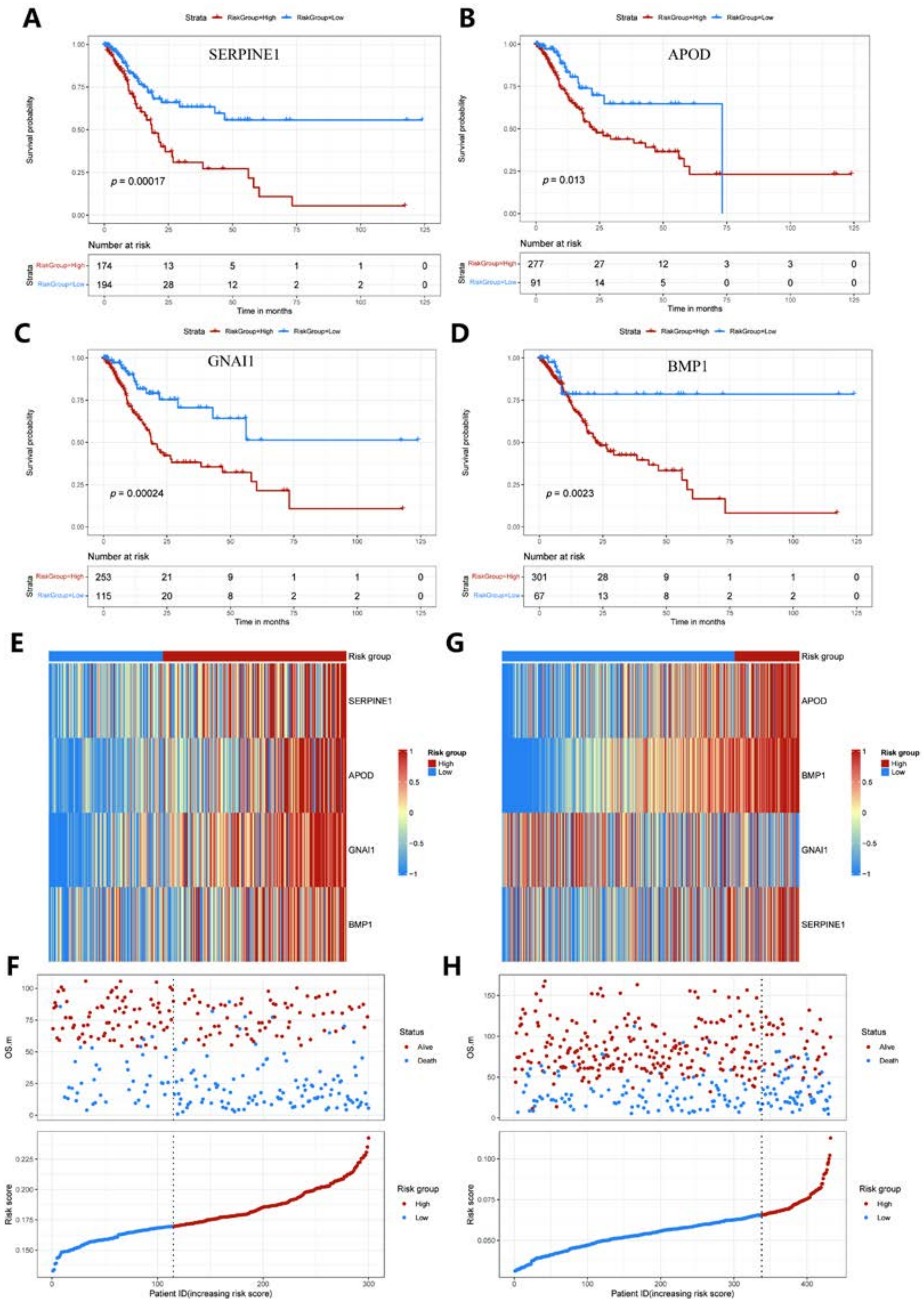

Figure S1. Construction of the IRS. (A-D) Kaplan-Meier OS analysis of four enrolled genes. (E, G) Heatmap of the IRS consisting of four IRGs in GSE62254 and GSE26253 cohorts. (F, H) Distribution of risk score, survival time, and status of patients in GSE62254 and GSE26253 cohorts.

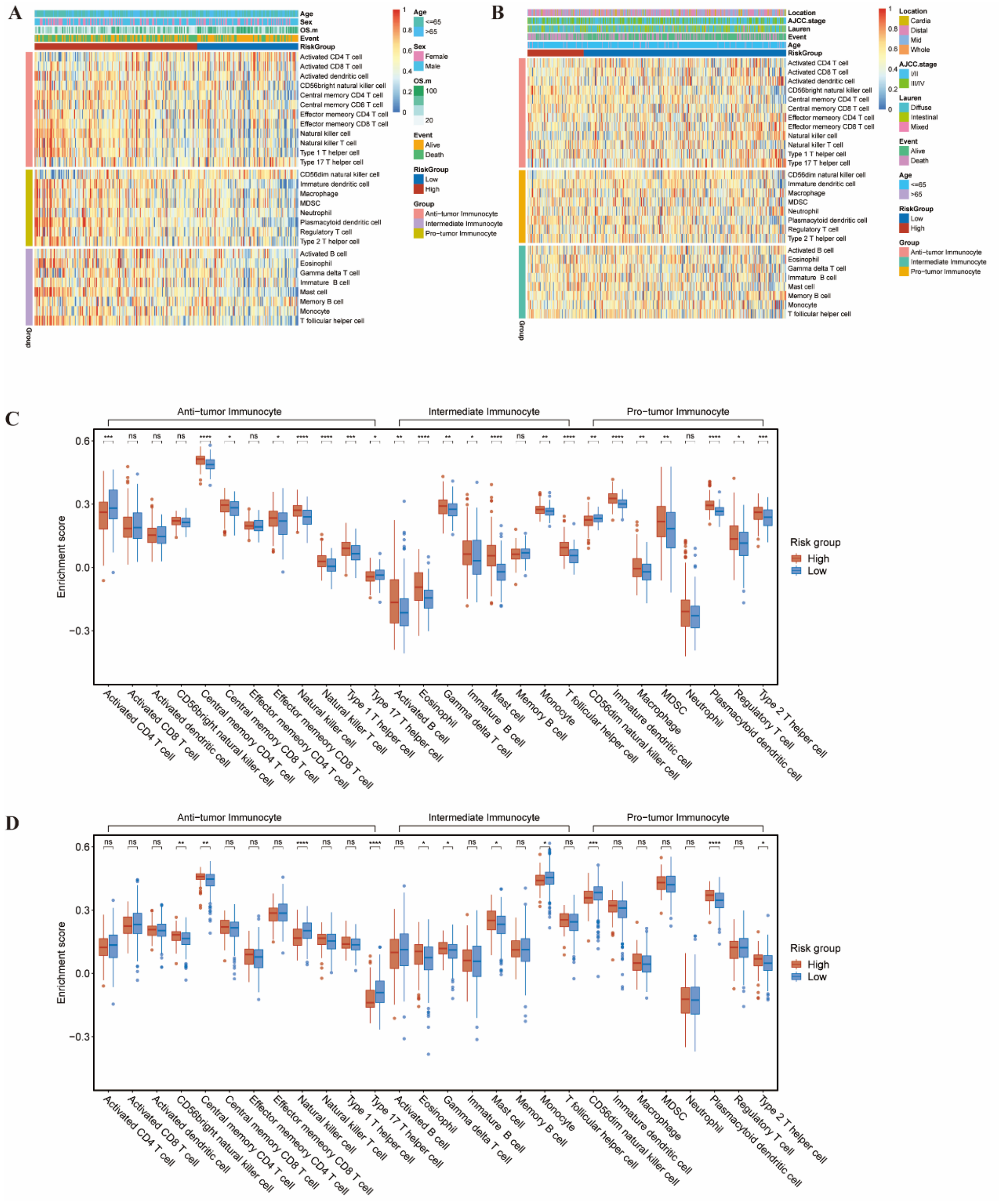

Figure S2. Transcriptome traits and clinical characteristics of TME phenotypes in the GEO validation cohorts. (A, B) ssGSEA identified the relative infiltration of 28 tumor-infiltrating immune cell types with different risk groups in GSE62254 and GSE26253 cohorts. (C-D) The relationship between risk score and 28 tumor-infiltrating immune cell types in GSE62254 and GSE26253 cohorts. Wilcoxon test, $^{*}p < 0.05$; $^{**}p < 0.01$; $^{***}p < 0{\cdot}001$; $^{****}p < 0.0001$; ns, not statistically significant.

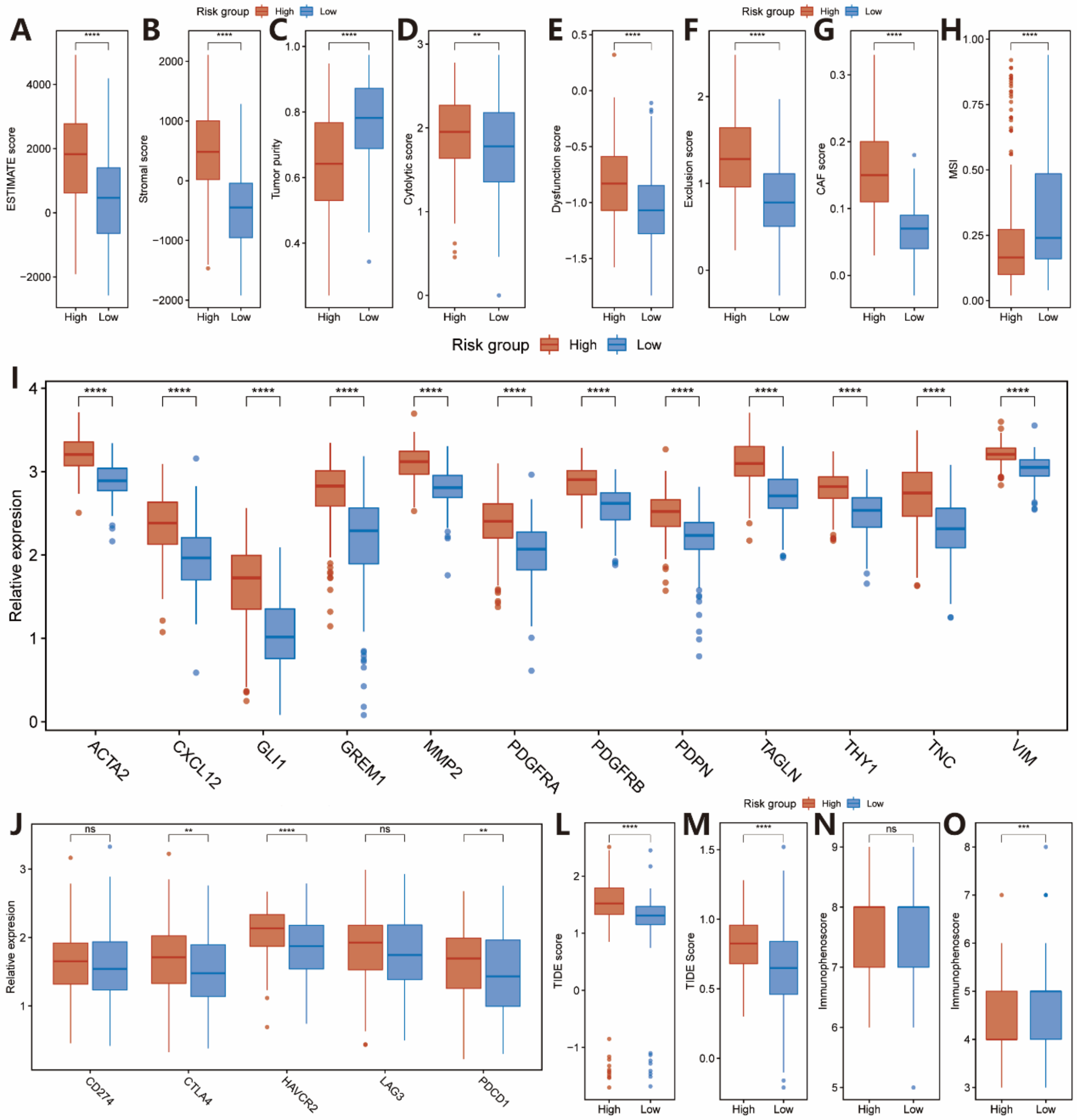

Figure S3. Biological function analysis between high- and low-risk groups in the three cohorts. (A-J) Boxplots for the comparison of ESTIMATE, stromal, tumor purity, cytolytic, T cell dysfunction, T cell exclusion, CAFs and MSI scores between two risk groups in TCGA-STAD cohort, followed by the relative expression of marker genes in CAFs and T cell exhaustion. (L-O) Boxplots of TIDE score and IPS between two risk groups in GSE62254 (L, N) and GSE26253 (M, O) cohorts. Wilcoxon test, $^{*}p < 0.05$; $^{**}p < 0.01$; $^{***}p < 0{\cdot}001$; $^{****}p < 0.0001$; ns, not statistically significant.

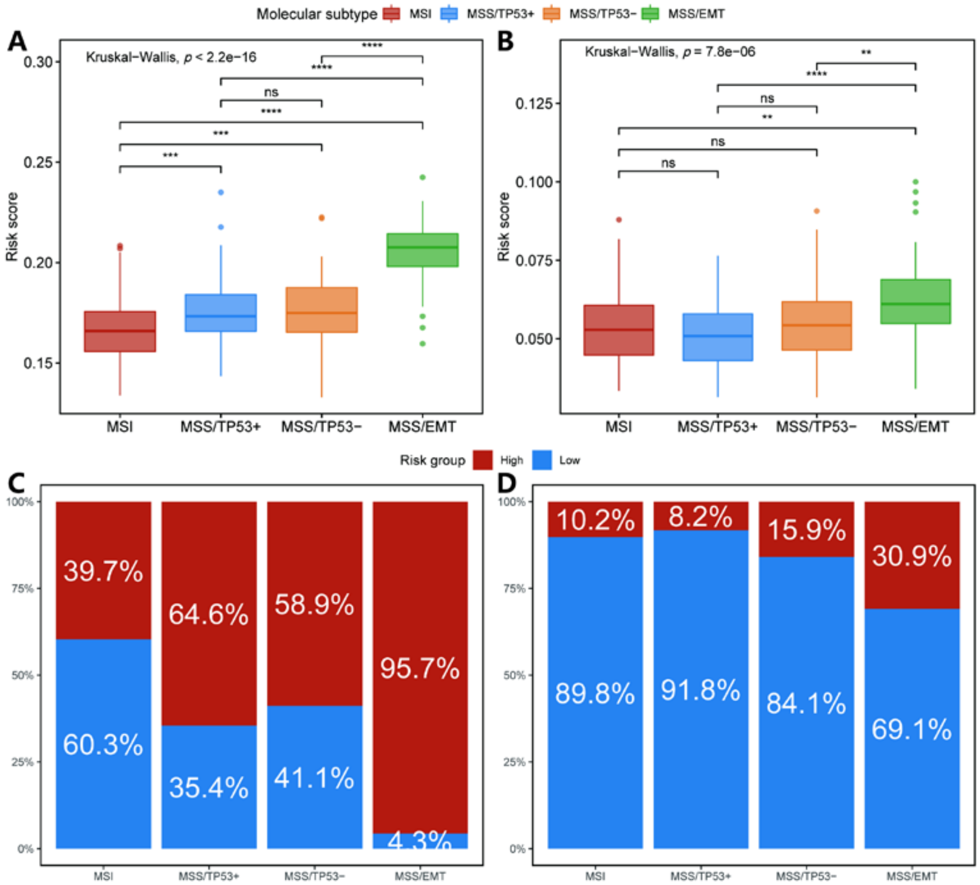

Figure S4. Relationship between risk score and ACRG molecular subtypes in the GEO validation cohorts. (A-D) Distribution of the risk score and the proportion of high-risk group in different ACRG molecular subtypes of the GSE62254 (A, C) and GSE26253(B, D) cohorts. Steel-Dwass test, $^{**}p < 0.01$; $^{****}p < 0.0001$; ns, not statistically significant.

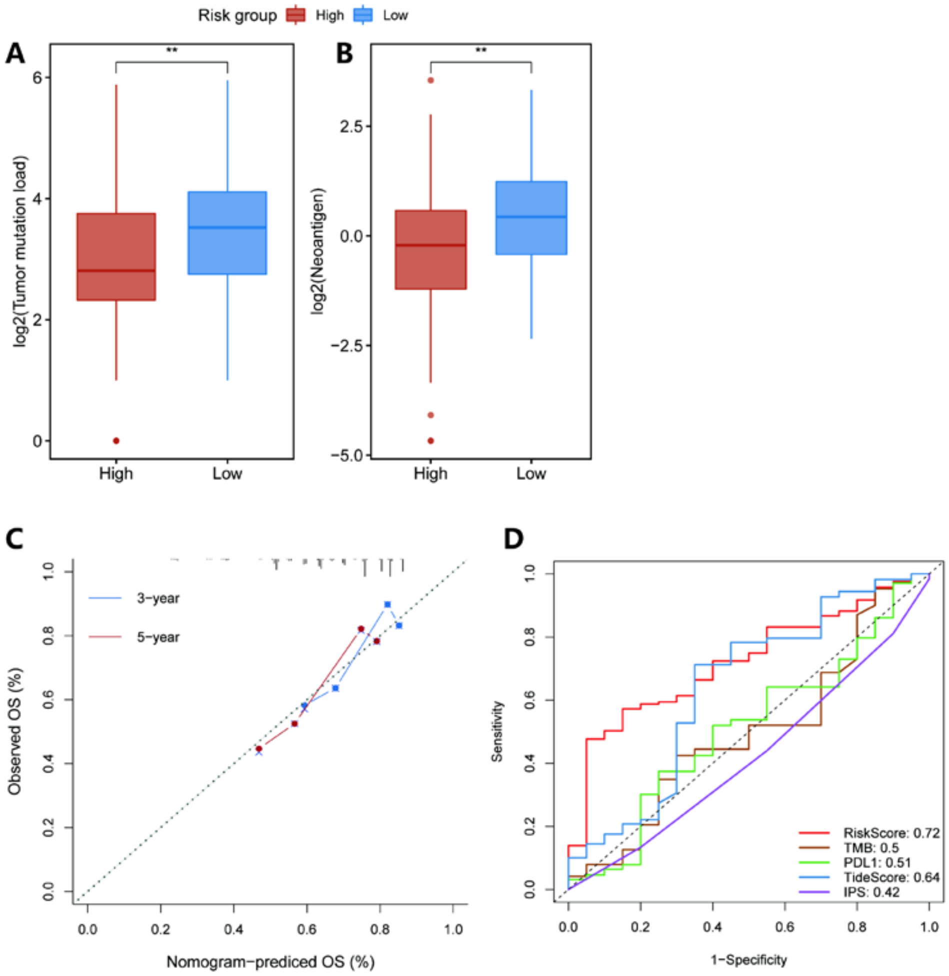

Figure S5. (A-B) TML and Neoantigen differences in the high- and low-risk groups of the IMvigor210 cohort, Wilcoxon test, $**p < 0.01$. (C) Calibration plot for predicted 3-year and 5-year OS in the GSE26253 cohort. (D) AUC for the risk score, TML, PD-L1 expression level, TIDE score, and IPS with OS for TCGA-STAD cohort.
